# Sedentary Behavior Research in the Chinese Population: A Systematic Scoping Review

**DOI:** 10.3390/ijerph17103576

**Published:** 2020-05-20

**Authors:** Ran Bao, Si-Tong Chen, Yanlei Wang, Jun Xu, Lei Wang, Liye Zou, Yujun Cai

**Affiliations:** 1 School of Physical Education and Sport Training, Shanghai University of Sport, Shanghai 200438, China; baoryan1955@gmail.com (R.B.); xujun050396@163.com (J.X.); wl2119@163.com (L.W.); 2 Institute for Health and Sport, Victoria University, Melbourne 3000, Australia; sitongchen@szu.edu.cn; 3 Harbin Institute of Physical Education, Harbin 150006, China; wyl0789@163.com; 4 Exercise and Mental Health Laboratory, Shenzhen Key Laboratory of Affective and Social Cognitive Science, Shenzhen University, Shenzhen 518060, China; LiyeZou123@gmail.com

**Keywords:** public health, sedentary behavior, China, review

## Abstract

*Background*: The negative effects of sedentary behavior (SB) on public health have been extensively documented. A large number of studies have demonstrated that high prevalence of SB is a critical factor of all-cause mortality. Globally, the frequency of SB research has continued to rise, but little is known about SB in the Chinese population. Therefore, this review was conducted to scope the research situation and to fill the gaps related to the effects of SB in the Chinese population. *Methods*: Using a scoping review based on York methodology, a comprehensive search of published journal articles and grey literature was carried out through 12 databases. The literature research was conducted by two authors in July 2019, and included journal articles that targeted on the Chinese population were published between 1999 and 2019. The two authors screened the records independently and included those research topics related to SB in the Chinese population. *Results*: The number of included studies increased from 1 to 29 per year during the analyzed period, during which, a remarkable climb happened from 8 in 2013 to 19 in July 2019. Out of the 1303 screened studies, a total of 162 studies (81 English and 81 Chinese journal articles) met the inclusion criteria in this review. Most of the included studies (66.0%) reported the overall estimated prevalence of SB, in which, 43.2% of studies reported the average time of SB, and 40.0% of studies reported the cutoff point of SB. Besides this, 54.9% and 23.5% of studies focused on the outcomes and correlates/determinants of SB, and the proportions of studies based on testing the validation of measurement tools and on interventions were 3.7% and 4.9%, respectively. Nearly all of the reviewed articles used data from cross-sectional studies (75.9%) and longitudinal studies (13.6%), while intervention trials are less developed. The majority of the studies (64.8%) used self-reported surveys, and only 3.7% studies used device-based measurement tools. Furthermore, 35.8% of the included studies were focused on children and adolescents, while only a few studies investigated infants/toddlers and older adults. Both female and male were examined in most studies, and non-clinical populations were investigated in the context of SB in a relatively large number of studies. *Conclusions*: The number of research articles on SB in the Chinese population published per year has increased year by year, indicating a growing interest in this research area. More studies using population subgroup samples are needed, particularly among infants/toddlers, older adults, and clinical populations. To provide stronger evidence of the determinants and outcomes of SB, longitudinal studies using device-based measures of SB are required.

## 1. Background

Sedentary behavior (SB) is highly prevalent in populations across the world [1]. However, from the perspective of human biological evolution, the human physiological structure is more suited to exercise [2]. Therefore, reducing SB (e.g., prolonged sitting or screen time) has become a worldwide public concern. SB can be defined as any waking behavior characterized by an energy expenditure of ≤1.5 metabolic equivalents (METs) while in a sitting, reclining, or lying posture [3], which has been recognized as a major risk to public health [4,5]. Prolonged SB is an important factor of health in various populations, such as increasing the rate of mortality [6,7], causing cardiovascular diseases [6,8], diabetes mellitus [9,10], metabolic syndrome [11], and cancer [12,13], impairing cognitive function [14], and triggering increased obesity [15]. Despite well-established evidence on detrimental effects of SB on population health, a high prevalence of SB has still been recorded in the literature over the past decades [2]. The alarming prevalence of SB has provoked global actions and initiatives to prevent the global trend. The World Health Organization (WHO) has reported that global mortality due to non-communicable diseases (NCDs) increased from 64% in 2000 to 71% in 2016 [16]. Based on the latest evidence, the mortality caused by cardiovascular diseases has increased by 20.9% over the past few decades [17], and overweight [18] and obesity remain highly prevalent and show an increasing trend in China [19]. Moreover, the burden of chronic NCDs has continued to increase in the past three decades, especially among the older adults in the Chinese population [17].

As the biggest developing and most populous country, China has suffered the burden of excessive SB. Many studies have shown a positive relationship between SB and NCDs [20] in the Chinese population, such as increased obesity [21,22], cardiovascular diseases [23,24], diabetes [25,26], cancer [27], and hypertension [28]. However, the data show that the Chinese population, regardless of gender and age, are increasingly exposed to extended SB [29,30,31]. For example, an increasing trend was found among Chinese children [32], and 30% of youths spent more than 3 h per day engaged in SB [31]. According to the Physical Activity and Fitness in China—The Youth Study (PAFCTYS), a large-population school-based survey [33], over 36.8% of students spent more than 2 h per day engaged in SB in 2016 [34], and 34.6% in 2017 [35]. Additionally, a high prevalence of SB has also been found in adults and older adults [24,30,36,37].

The higher levels of SB in the Chinese population are mainly due to the rapid change of society [38]. Evidence has shown that the built environment is associated with excessive SB [39]. In China, a sharp increase in the urban population has resulted in a shortage of public resources (e.g., insufficient exercise facilities [40]) and an obesogenic environment (e.g., more sitting) [41]. These changes may be the cause of the high prevalence of SB and NCDs in China [38]. On the other hand, the rapid change in the structure of the population is another great problem that China has to cope with, particularly in terms of the subsequent rise in SB. In regard to size, China’s aging population is the fourth largest worldwide [42]. In comparison, although China entered an aging society later than Japan, the process of aging is faster and starts earlier for the Chinese population [43]. In addition, social culture may be another reason for the high level of SB. For instance, Chinese youths face more social and cultural pressure related to academic performance [44,45]—the length of time Chinese children and adolescents spend on doing their homework is greater than that of children and adolescents in Canada and the UK [44].

Faced with such concerning public health issues, the Chinese government has issued some initiatives and actions to minimize the health burden and to resolve the related health problems. The recent Healthy China 2030 Blueprint stresses that promoting an active lifestyle in the Chinese population is the biggest health plan action for achieving improved health [46]. Other similar actions for younger people, such as the National Fitness Program (2016–2020) and the Youth Sports Promotion Program (2017), have also established national-level policies for reducing SB among this population, which have been supported by policymakers.

Over the past decade, epidemiological SB research has been on the rise, owing to its detrimental effects. In this field, many previous systematic reviews have examined the prevalence, correlates, and health outcomes of SB across different populations. However, those reviews are narrow in nature, focusing on specific domains in the field of SB research, which in turn limits researchers’ insights into the populations in some countries. The deficiency may result in an imbalance of SB research across the world. Also, there is no comprehensive review that investigates studies of SB among the Chinese population. Indeed, addressing or preventing the high prevalence of SB in the Chinese population would provide complementary benefits to reduce the global health burden of excessive SB. Hence, our study aims to examine the studies on SB among the Chinese population by using a scoping systematic approach. In turn, this study identifies gaps in the SB research in the Chinese population, and proposes future recommendations and directions for epidemiological SB research and policy practice.

## 2. Methods

### 2.1. Search Strategy

This scoping review was performed based on the York methodology outlined by Arksey and O’Malley [47,48], and according to the Guidance for Conducting Systematic Scoping Reviews [49]. With the aim of identifying research gaps and mapping out the existing literature by examining the extent and nature of said research, this review was conducted as a scoping review. A literature search was conducted to identify relevant published studies about SB in the Chinese population. Before the literature search, we discussed the details about the research question and the search strategy. Then, searches were conducted in 12 electronical databases (WOS, PubMed, EBSCO, Academic Search Premier, SPORTDiscus, MEDLINE Complete, Psychology and Behavioral Sciences Collection, ScienceDirect, ERIC, PsycINFO, SCOPUS, CNKI, and WANGFANG DATA) in July 2019. The definition of sedentary behavior in this review refers to a sitting or leaning posture with an energy consumption less than 1.5 METS during any waking behavior [50]. The search terms were discussed by the authors, and the keywords included “sedentary behavior” or “sitting time” or “screen time.” Appendix A shows the detailed search strategies used for each database. The search was modified as needed to meet the different database’s search criteria. Additional articles and grey literature documents were identified via a literature search of the reference lists of all of the articles selected in the database search.

### 2.2. Study Selection and Inclusion Criteria

Two authors separately conducted the literature review, and then combined and removed duplicated literature. All records from the literature search were imported into EndNote X9 software. After removing duplicates, the remained records were screened independently by two reviewers in terms of their titles and abstracts (BR and XJ). Any disagreements between the two reviewers were resolved in discussion, and a consensus was reached with a third reviewer (SC).

Studies were included in the present review if they: (1) included any Chinese population in China; (2) conducted research on SB, including any waking screen or sitting behavior characterized by an energy expenditure ≤ 1.5 metabolic equivalents (METs) in daily life; (3) collected any quantitative data related to SB, including levels, prevalence, correlates, determinants, outcomes, measurement tool or intervention (intervention trials in the Chinese population in China) studies; (4) included any type of SB measure, such as self-reports or device-based measures; and (5) published in either a English or Chinese language peer-reviewed journal article. Studies were excluded if they: (1) targeted non-Chinese populations and Chinese people outside of China; (2) the main outcome(s) focused on sports/exercise/skill performance or physical therapy; or (3) were published as a literature review, commentary, conference abstract, or editorial.

### 2.3. Data Extraction

The data of the included studies were compiled according to the following data extraction method, and descriptive analysis and reporting frequency counts were performed. The purpose of the review was discussed, taking into account the impact on research, policy, and practice.

The following data were extracted from the included studies: (1) administrative information, including author names, publication year, and title; (2) the study method, including the study design, survey method, sample size, and sampling method; (3) characteristics of the study sample, including sex, age, municipality (rural/urban), region, and other specific characteristics of the participants; (4) the study measure, including the type of SB measure, the device or questionnaire name, the type of SB (such as sitting time, screen time, and transport), the validity of measures, and the intervention type); and (5) the study objective, including determinants/correlates, and outcomes topics categorized according to previous studies [15,51,52]. Definitions of these topics are as followed: (1) Socio-demographic includes age, sex, place of residence, religion, educational level, membership in organizations and social groups; (2) genetic includes gene-related indicators; (3) health behaviors/lifestyle/knowledge include physical activity, life habits, health knowledge; (4) general health includes overweight/obesity, sleep quality, fat mass, and other health indexes; (5) diseases mean chronic noncommunicable diseases, such as, cancer, diabetes, hypertension, cardiovascular; (6) skills/abilities/fitness include sport skill, body abilities and fitness; (7) psychological factors include mental health, cognitive ability and others; (8) social and culture include social device or feeling support; (9) built/natural environment includes those built or natural environment affecting SB; (10) academic performance includes academic burden or pressure. The detailed data extraction table for the studies used in this review is available in Additional file 1.

## 3. Results

**Figure 1:**
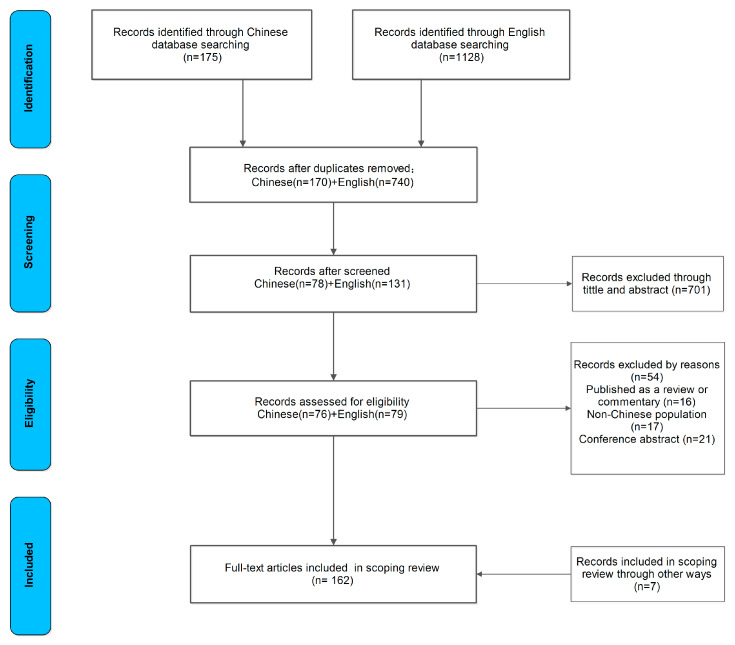
Flow diagram of the study selection process.

The literature search and selection processes can be found in Figure 1. In total, 175 Chinese and 1128 English journal articles were identified through the database search, of which 170 Chinese and 740 English records remained after removing duplicates. Following title and abstract scanning, 78 Chinese and 131 English potentially relevant articles were retained. According to the inclusion and exclusion criteria, 76 Chinese and 79 English full-text records were included, and seven more articles were added from reading those full-text records. Finally, a total of 162 studies were included for this review [20,21,22,23,24,25,26,27,28,29,30,32,34,35,36,37,44,53,54,55,56,57,58,59,60,61,62,63,64,65,66,67,68,69,70,71,72,73,74,75,76,77,78,79,80,81,82,83,84,85,86,87,88,89,90,91,92,93,94,95,96,97,98,99,100,101,102,103,104,105,106,107,108,109,110,111,112,113,114,115,116,117,118,119,120,121,122,123,124,125,126,127,128,129,130,131,132,133,134,135,136,137,138,139,140,141,142,143,144,145,146,147,148,149,150,151,152,153,154,155,156,157,158,159,160,161,162,163,164,165,166,167,168,169,170,171,172,173,174,175,176,177,178,179,180,181,182,183,184,185,186,187,188,189,190,191,192,193,194,195,196,197].

### 3.1. Description for Trends of the Included Studies

**Figure 2:**
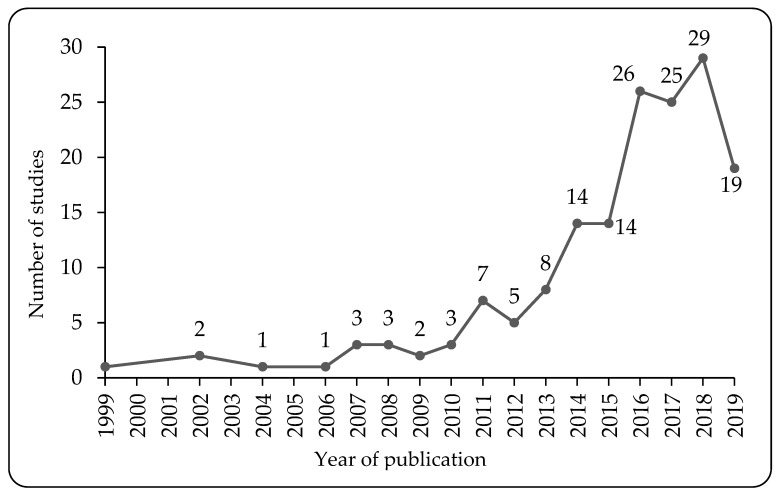
The number of studies on SB published per year (time to July 2019).

All articles included in this review were published between 1999 and 2019, the details of which are shown in Figure 2. As can be seen, the number of papers published per year has witnessed an increasing trend over the analyzed period. The number of SB-based articles focusing specifically on the Chinese population increased steadily from 1999 to 2013, then climbed sharply during the following 5 years, with a peak at 29 in 2018. Although the literature search was conducted in July of 2019, the number of SB-related studies in 2019 was still high.

### 3.2. Characteristics of the Study Design and the Sample

The characteristics of the study design and the sample are outlined in Table 1. Of the studies included in this review, most of the quantitative studies were cross-sectional surveys (75.9%) aimed at detecting the relationship between SB and its correlates or outcomes. However, few studies were of a longitudinal design (13.6%), and only 4.9%, 3.1%, and 2.5% were intervention trials [54,103,118,136,137,138,194,195], measurement studies [64,75,108,119,125], or case-control studies [21,78,84,113], respectively. The sample sizes of the included studies ranged from 26 to 1,048,594, and 20.4% of the studies were conducted using nationally representative samples. Of these studies, 63.6% solely conducted secondary data analyses of national surveys, such as the Physical Activity and Fitness in China—The Youth Study, the China Health and Nutrition Surveys (CHNS), the China Education Panel Survey–Junior High Cohorts study, the 2014 Physical Fitness and Health Index of Child and Adolescents (PFHICA) study, and the Chinese Youth Risk Behavior Survey. Most studies (45.1%) were conducted in urban regions, followed by 27.2% in both urban and rural regions, and only 3.1% in rural regions, the details of which are shown in Table 1.

Participants of both genders were included in 87.0% of the studies. Studies of females only (5.6%) were more common than studies of males only (2.5%). Only children and adolescents (7–17 years) were the most frequently investigated age group (35.8%), followed by adults (18–64 years; 11.1%), infants/toddlers (3–6 years; 3.1%), and older adults (≥65; 1.9%). The large majority of the studies were conducted in non-clinical populations (88.9%), of which, 46.9% were conducted among primary school, secondary school, high school, and university students. The remaining studies targeted the general population (35.8%), patients (7.4%), the occupational population (3.1%), or others (6.2%), the details of which are shown in Table 2.

### 3.3. Measurement of Sedentary Behavior

**Figure 3:**
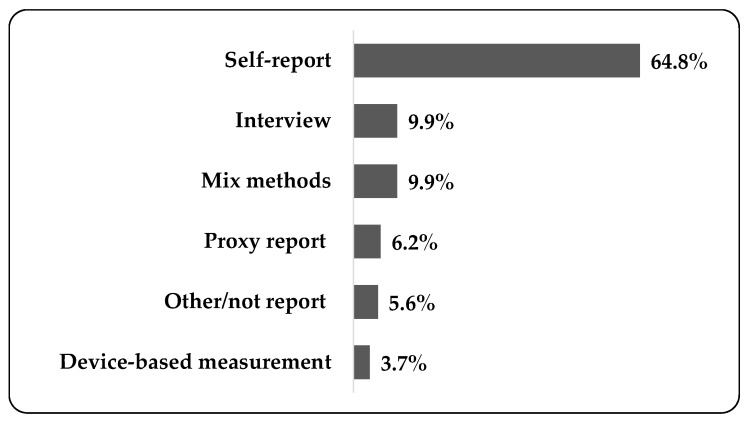
The proportion of data collection methods of SB (mixed method: the use two or more methods, e.g., a questionnaire and an accelerometer).

The details of the data collection methods are shown in Figure 3. The majority of the included studies (64.8%) used questionnaire-based self-report measures; 49.4% of the studies did not specify the type of questionnaire; International Physical Activity Questionnaires (IPAQ) and accelerometers were used in 14 (8.6%) and seven (4.3%) studies, respectively; the Youth Risk Behavior Surveillance System (YRBS) questionnaire and the Health Behavior School-aged Children (HBSC) questionnaire were used in four and two studies, respectively; and 27.8% of the included studies reported the validity of the questionnaire(s) used. Furthermore, interviews and mix methods (the use two or more methods, e.g., a questionnaire and an accelerometer) were used in 16 studies (9.9%); a proxy report was used in 10 studies (6.2%), including a parent/guardian or teacher proxy; nine studies (5.6%) used other methods (e.g., the inquiry method) or did not report a method; and only six studies (3.7%) used device-based measures, including an accelerometer or a pedometer.

### 3.4. Categorizations of the Study Topics

**Figure 4:**
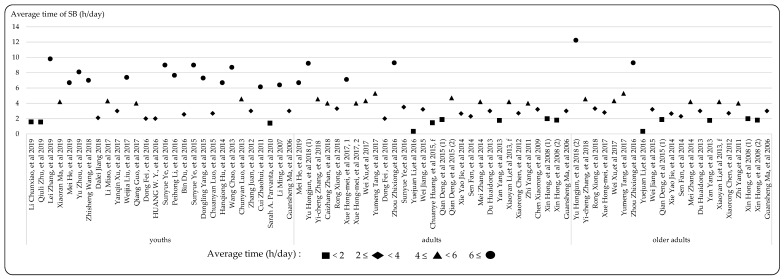
The average duration (h/day) engaged in SB (in the form [198]) (abbreviations: m = male; f = female; youths < 18 years; 18 years ≤ adults < 65 years; older adult ≥ 65 years; Xue Hong-Mei, et al. 2017, 1 = 20–40 years [196]; Xue Hong-Mei, et al. 2017, 2 = 41–55 years [196]).

**Figure 5:**
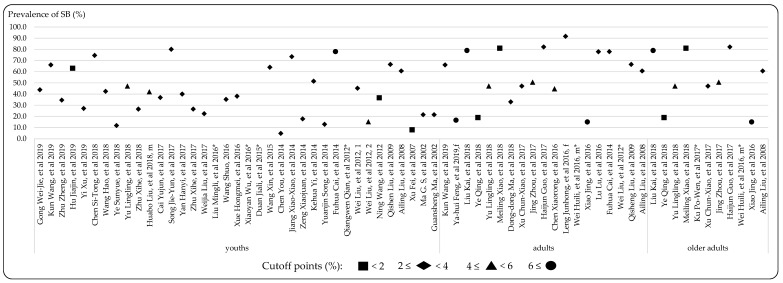
The prevalence (%) of SB of included studies reporting the cutoff points (in the form [198]) (abbreviations: * No information on overall prevalence available; m = male; f = female; youths < 18 years; 18 years ≤ adults < 65 years; older adult ≥ 65 years; Wei Liu, et al. 2012, 1 = screen time [177]; Wei Liu, et al. 2012, 2 = sitting time [177]).

In the studies included in this review, a substantial proportion (66.0%) reported the estimated prevalence of SB. Specifically, 28.4% focused on both screen time and sitting time; 56.1% reported screen time, including TV viewing, computer use, videogames, and internet/social networking; and 40.1% concentrated on sitting time, including reading, writing, drawing, homework, and playing musical instruments. However, only five studies reported transport-related SB. Moreover, in these prevalence studies, 43.2% reported the duration of SB, the details of which are outlined in Figure 4. Specifically, 32.1% of studies demonstrated that participants spent between 2 to 4 h per day engaged in SB, while duration of 4–6 h and over 6 h per day were reported in 22.6% and 28.3% of the studies, respectively. Most studies reported that youths spent more than 2 h per day, varying from 1.55 to 8.7 h per day engaged in SB, while 41.4% of studies reported that youths spent more than 6 h per day engaged in SB. In terms of adults and older adults, 10.7% and 9.5% of studies reported that more than 6 h per day was spent engaged in SB, respectively. In addition, 40.0% of the studies reported the cutoff points to detect the prevalence of SB, the details of which are outlined in Figure 5. Specifically, 62.0% of the studies reported the proportion of participants who spent more than 2 h per day engaged in SB, while 6.0% and 10.0% reported more than 4 h and 6 h, respectively. The different prevalence results are related to the diversity of the cutoff point; 70.3% of the studies that used cutoff points (≥2 h per day) reported the percentage of youth in sedentariness, ranging from 4.7% to 80.0%. Meanwhile, in terms of adults and older adults, with the proportion reporting SB ranging from 15.0% to 93.5%.

Details of the correlates/determinants and study outcomes (e.g., genetic, health variables) of SB are outlined in Table 3. Of the included studies, the general aims focused on the outcomes of SB (54.9%). The main aims of the outcome studies were focused on general health (39.4%), such as obesity, overweight, sleep quality and others; disease (35.4%), including chronic diseases, morbidity, diabetes, cancer and others; psychological factors (11.1%), such as, depression, anxiety symptoms, cognitive ability, and psychiatric symptoms. Genetical factors, health behaviors, and skills/abilities/fitness were seldom identified in the Chinese population.

In total, the proportion of studies concentrated on the correlates/determinants of SB was 24.1%. Among these studies, the socio-demographic factors (34.6%) were the most common correlates, such as age, gender, and education level, followed by health behavior/lifestyle/knowledge factors (17.9%), such as physical activity, diet, and health knowledge; 17.9% of the studies concentrated on social and cultural factors, such as social support, parents’ rules, and parental modeling, sport organization; and 12.8% of the studies focused on the built/natural environment, such as physical activity facilities, the weather, safety of and/or access to public transport. A limited number of studies (11.5%) investigated psychological (e.g., attitude, self-efficacy), academic performance, skills/abilities/fitness factors.

Moreover, few studies targeted the validation of an SB-related measurement tool (3.7%) or of intervention trials (3.8%). Only two of them tested the validation of the Health Behavior in School-aged Children (HBSC) survey questionnaire and the long-term and short-term recall of occupational sitting time questionnaire, and one focused on the Sense Wear Mini Armband.

## 4. Discussion

This scoping review examined and summarized the evidence of SB research in the Chinese population. Firstly, literature review indicated that the number of SB research in the Chinese populations increased rapidly over the past two decades, and 71% of the included studies were published over the past five years, which is consistent with the figures reported by other countries [52,199]. This increasing trend demonstrates that SB has become a popular research topic in China. Secondly, the included studies reported that Chinese population spent a large amount of time on SB. Moreover, socio-demographic, health behaviors/lifestyle/knowledge, cultural/social, and built/ natural environment factors were the main aspects of correlates of SB, and general health and diseases were the main outcome topics of the relevant studies in this review. Thirdly, the majority of the included studies targeted children and adolescents using cross-sectional design and self-report questionnaires. In addition, most of the empirical research studies investigated the specific types of SB, including screen time (such as watching TV, using computer/internet, playing electronical games) and sitting time (such as doing homework, playing musical instruments, reading/writing). Only a limited number of studies focused on transport-related SB.

### 4.1. SB Study Design and Measurement

The majority of the studies in this review were cross-sectional design and used self-report measures, which is consistent with previous similar reviews [200,201,202]. Time and cost burden may be a barrier of conducting longitudinal and interventional studies in China. However, cross-sectional studies cannot provide the cause-and-effect association between exposures (i.e., independent variables) and outcomes (i.e., dependent variables). More longitudinal studies are encouraged to conduct in the Chinese population. In addition, several studies included in this review were based on national surveys, such as the Physical Activity and Fitness in China—The Youth Study (PAFCTYS), the China Health and Nutrition Surveys (CHNS), and the China Education Panel Survey–Junior High Cohorts study. Notably, few studies targeted younger children (e.g., 3–6 years) and older adults (e.g., ≥65 years). To improve the generalizability of the findings from SB epidemiolocal studies, more studies should be encouraged to focus on special populations in the future.

In terms of the measurement of SB in this review, most of the included studies used self-report methods to collect data. Although self-reported measures (e.g., questionnaires) are susceptible to low response rates, misclassifications, and difficulty in determining the direction of the effects between variables [203], this method is still widely used in SB research at large-scale level [204,205]. Although questionnaires were used by the studies included in this review, such as the IPAQ, the Children’s Leisure Activities Study Survey questionnaire—Chinese version, and the Youth Risk Behavior Survey System (YRBSS) questionnaire, only half of these studies reported the validity. In order to ensure the reliability and validity of data, the use of questionnaires with accepted reliability and validity is mandatory. In contrast, device-based measures (e.g., accelerometers, pedometers) can provide more accurate data; however, such measures only mechanically record moving or acceleration information of the wearing section of body, and fail to identify specific body postures and types of SB (e.g., whether carrying weight or not, standing, or sitting) [206]. Although device-based measurements have many limitations when evaluating a particular type of SB, compared to different modified versions of the questionnaire, it is easier to obtain validity in international comparisons [204]. Currently, posture monitors such as activPAL have become a reliable and valid measure in recording SB patterns [207,208]. One study even suggests that combining movement monitors (accelerometers) with physiological sensors (heat rate monitors) might be a potential method to improve the accuracy of SB measurement [209]. Therefore, for a better understanding, there is a need for more studies to use valid questionnaires and device-based measures to collect SB data in the Chinese population. In addition, the majority of the included studies focused on screen time, such as TV viewing, computer use, videogames, and internet/social networking, but failed to distinguish between the different outcomes of screen time, traffic SB, and recreational SB. Furthermore, evidence has demonstrated that different types of SB generate differentiated health risks. For example, SB in regard to television viewing is related to cardiovascular disease, but the same results are not found when using a computer [210]. Therefore, investigating the effects of different types of SB on public health can provide a more effective mechanism for reducing SB itself.

### 4.2. SB Sub-Populations

Youths and adults were the most commonly studied sub-populations, followed by mixed groups (more than two age groups)/not reported, and then adults (18–64 years); few studies targeted infants/toddlers or older adults. This is in contrast to a study in Thailand, which indicated that adults (18–59) are the most commonly studied age group [52]. The main reason might be the convenience in measuring and detecting the detrimental effects (e.g., chronic diseases) of SB in middle-aged adults. Besides, another reason may be that the Chinese government is greatly concerning about youth health. Due to the severe health problems of children and adolescents [211], the Chinese government has been spending a lot of manpower and resources to overcome this public health issue. Obviously, the orientation of policy has affected on the popularity of choosing children and adolescents as targets. In addition, the difficulties related to conducting surveys with infants/toddlers and older adults may also partially explain why most Chinese SB studies focus on younger age groups. Therefore, more research into these age groups is required in the future.

Moreover, the majority of the included studies concentrated on student and general populations, but few focused on the occupation-specific population, the obese/overweight population, pregnant and postpartum women, and religious and other populations in China. Recently, studies have revealed that maternal recreational exercise during pregnancy is related to 7-year-old children’s body mass index (BMI) scores and their risk of becoming overweight [212]; SB during pregnancy is also reported to generate a negative impact on pregnant women’s health [213]. However, whether SB during pregnancy really does have negative impacts on the health of women and infants requires more evidence. Nowadays, insufficient awareness of the health risks associated with SB has resulted in a rapid increase in the prevalence of obesity and chronic diseases in Chinese society, which has resulted in concerning medical burdens for society. The death rate as a result of chronic diseases in middle-aged people is higher in China than in various high-income countries [214]. As the rate of the aging population, of urbanization, and of social and economic reforms continues to progress, the prevalence of chronic diseases in China will continue to rise. Therefore, researchers should pay more attention to SB of special populations in China.

### 4.3. SB Study Topics

Notably, a high prevalence of sedentariness was not only found in Chinese youths, but also in adults and older adults. However, due to the lack of consistent standards, it is hard to extract the overall estimated prevalence of SB in population subgroups. Most of the prevalence studies concentrated on screen time and sitting time, while a limited number of studies focused on transport-related SB. However, SB related to transport has been widely examined in other countries, the results of which show differences between urban and rural environments. Furthermore, one study has indicated that SB may be replaced by standing or slow movement in urban transport settings [215]. Therefore, further studies should consider refining transport-related SB research. Moreover, studies have shown that the effects of different volumes or types of SB may result in a variety of health outcomes [216]. In this review, although most of the included studies targeted the prevalence of SB, few studies focused on the prevalence of SB within different locations and of types. Additionally, it was observed that the cutoff points of SB vary from 2 to 9 h per day, although most studies used 2 h per day to assess the estimated prevalence of screen time for the Chinese population in this review. The average time youths spent on SB was indicated to be more than 6 h per day. Furthermore, different amounts of SB cause a diversity of health risks in different age groups, and a lack of risk criteria related to SB for specific populations may make it difficult to accurately predict health risks of said population groups [217].

The research topics included in this review encompassed multiple dimensions, which is consistent with current SB research around the world [218,219,220,221]. The studies included in this review mostly concentrated on the outcomes of SB, while few studies focused on the determinants/correlates. General health problems (e.g., obesity, overweight, sleep quality) and diseases (e.g., cardiovascular, metabolic, and mental diseases) were the main topics of the outcomes in this review. Due to insufficient evidence, it is hard to determine whether other diseases such as cancer, type 2 diabetes, and musculoskeletal disorders also have a certain relationship with SB [15]. It is worth noting that the prevalence of diabetes and cancer has continuously increased in recent years in China [222,223], and a quarter of diabetes patients around the world were located in China [224]. More studies are encouraged to focus on these diseases, especially in adults and the aging population in China. Meanwhile, a limited number of studies have concentrated on genetical factors, health behaviors, or skills/abilities/fitness factors. For example, it has been revealed that screen (TV viewing) and sitting time is related to unhealthy behavior (e.g., physical inactivity, diet), including aggression in children and smoking or alcohol consumption in adults [15]. Another review indicated that SB is inversely associated with physically aggressive behavior in younger children, and most studies identified this relationship via TV viewing time [225]. Recently, issues related to violence in children have frequently occurred in China; however, few of the studies focused on the outcome of aggression in this review. Therefore, research into whether sitting time or other types of SB are related to other risk behaviors in Chinese youths is still required. In this review, a few studies focused on academic performance as a correlate, but none identified the effects of SB on academic performance in China. For instance, these studies showed that spending a long time engaged in SB is associated with decreased academic performance [226,227]; however, the relationship between spending more time on homework [44] and academic performance (e.g., cognition performance, grade point, memory, attention) [226] is not clear in Chinese youths. In future research, the relationships should be clearly clarified.

In this review, the determinants/correlates of the Chinese population can be divided into several main categories, including individual, social, and environmental factors [52]. Such categories include socio-demographic, health behaviors/lifestyle/knowledge, skills/abilities/fitness, psychological, academic performance, social and cultural, built/natural environment, which is consistent with the outcomes of two determinant studies [201,228]. However, the majority of included studies focused on socio-demographic (e.g., age, gender, education level), health behavior (e.g., exercise), and social/culture (e.g., social support) factors, and few studies focused on built environment, psychology, academic performance. In terms of academic performance, high social and cultural pressure may be an important explanation of the high prevalence of SB in Chinese youths. For example, evidence has shown that Chinese children spend more time than children from other countries (e.g., Canada, UK) on homework [44]; this relationship has been widely examined in other countries, the outcomes of which highlight the negative impact of SB on academic performance among children and adolescents [227]. In this review, however, only four studies focused on the relationship between academic performance and SB on Chinese youths. Further studies need to focus on this field in order to elucidate the relationship between academic performance and SB in China. Moreover, SB-related policies are undeveloped in China, with limited policies focusing on reducing SB. Previously, the orientation of policies to Chinese research has been discussed in this review, therefore, effective policies should be encouraged to reduce SB in the Chinese population. In addition, few of the studies included in this review concentrated on the built environment, and of those that did, most of them targeted children and adolescents, which is consistent with a different review [39]. Nowadays, changes of built environment (e.g., insufficient exercise facilities and obesogenic environment) and fast process of aging have been main challenges for limiting SB in the Chinese population. Further studies focusing on built environments should be encouraged in China, especially in adults and the elderly.

However, only eight studies were intervention trials in this review. This finding is consistent with several studies, which showed interventions for SB developed in Europe, the USA, and Australia, but not in China [229,230]. This may be related to limited evidence regarding the correlates for population subgroups. Most SB research are at individual level, but a single dimension is inefficient for addressing SB [231]. The socio-ecological model highlights the importance of multi-level factors in terms of addressing public health issues; thus, there is a dynamic relationship between the individual level, the intrapersonal level (i.e., social support [232]), the organization level, the community level, and the policy level [221]. In order to develop more effective behaviors reduction interventions, further studies need to concentrate on the dynamic relationship between these factors of SB in the Chinese population. In this way, more feasible and effective intervention programs can be conducted on the Chinese population based on these correlates [233].

### 4.4. Strengths and Limitations

The advantage of this review lies in its use of systematic search and selection strategies to identify relevant studies. Furthermore, a large number of sedentary behavioral studies on the Chinese population were retrieved through 12 English or Chinese electronical databases. Second, the determinants/correlates and outcomes were categorized to facilitate the development of follow-up research. However, there are some limitations to this scoping review. While it is desirable to include as many studies as possible that meet the inclusion criteria, there may be omissions due to the availability of the study and the comprehensive nature of the database. In addition, due to the limit of heterogeneity in SB (e.g., different types and measures of SB), this review focused on the description of the basic characteristics. In the future, the epidemiological characteristics of SB in the Chinese context should be summarized for different ages, different groups of people, and different study topics.

### 4.5. Recommendations for Future Research

Based on the aforementioned analysis, several suggestions can be made for directing future research conducted in China. Firstly, based on the obvious policy orientation of SB research in China, as well as the high prevalence of SB and chronic disease rejuvenation, future research should focus on the correlates/determinates of SB. Secondly, future research should combine the advantages of both self-report and device-based tools to improve the accuracy of SB measurement. Meanwhile, more longitudinal studies should be designed to detect the cause-and-effect relationship of SB. Future studies in China could also be strengthened by using validated device-based and self-report measures of SB. Additionally, for a better understanding of the determinants and outcomes of SB in China, future studies should aim to use longitudinal study designs. Thirdly, due to the differences of the determinates and outcomes between population subgroups, it might be useful to concentrate on specific population subgroups, such as infants/toddlers, occupation-specific population, older adults, or clinical populations. Finally, socio-demographic factors were the main focus in the current research in China, and more research is needed on academic performance, built/natural environment, psychology, and social/culture factors relevant to SB.

## 5. Conclusions

In the past decade, SB research has shown a spurt of development. The included studies in this review indicated the high prevalence and gaps of SB research in the Chinese population. However, SB research still has many limitations, such as the methodology used, research topics, and research populations, all of which hinder the healthy development of this research area in China. In further research, more studies should be conducted in the Chinese population subgroups, especially focused on developing more efficient strategies to reduce SB.

## Figures and Tables

**Table 1:** Characteristics of the study design and sample.

Study Design	Number	Proportion
Cross-sectional studies	123	75.9%
Longitudinal studies	22	13.6%
Intervention trials	8	4.9%
Measurement studies	5	3.1%
Case-control studies	4	2.5%
National surveys (secondary data analysis)	33 (21)	20.4% (63.6%)
**Sample gender**		
Female and male	141	87.0%
Female	9	5.6%
Male	4	2.5%
Not report	8	4.9%
**Population subgroup**		
Only Infants/toddlers	5	3.1%
Only Children and adolescents	58	35.8%
Only Adults	18	11.1%
Only Older adults	3	1.9%
**Sample region**		
Urban and rural	44	27.2%
Only urban	73	45.1%
Only rural	5	3.1%

**Table 2:** The population groups included in the studies on SB research of Chinese population.

Population Groups	No. of Studies
**Non-clinical populations**	
Students	76
General (no specific characteristics)	58
Occupation-specific populations	5
Health-care students	1
Obese/overweight	1
Multiple populations groups	1
High risks of chronic diseases population	1
Religious groups	1
Total	144
**Clinical populations (general characteristic)**	
Chronic/diabetes/hypertension	5
Pregnant and postpartum women	3
Cardiovascular	2
Patients (no specific characteristics)	1
Acute coronary syndromes	1
Epithelial ovarian cancer	1
Colorectal cancer	1
Esophageal squamous cell carcinoma	1
Male infertility	1
Total	16
Not reported	2
**TOTAL**	162

**Table 3:** Number of studies investigating the correlates and outcomes of SB.

Categories	Correlates		Outcomes	
	No. of Studies	%	No. of Studies	%
(1) Socio-demographic	27	34.6%	--	--
(2) Genetic	--	--	1	1.0%
(3) Health behaviors/lifestyle/knowledge	14	17.9%	5	5.1%
(4) General health	1	1.3%	39	39.4%
(5) Disease	3	3.8%	35	35.4%
(6) Skills/abilities/fitness	2	2.6%	8	8.1%
(7) Psychological factors	3	3.8%	11	11.1%
(8) Social and culture	14	17.9%	--	--
(9) Built/natural environment	10	12.8%	--	--
(10) Academic performance	4	5.1%	--	--
Total *	78(38)	100%	99(89)	100%

Note: * Multiple correlates and/or outcomes were investigated in some studies; hence, the sum of the totals is greater than the total number of these studies.

## Data Availability

The summary of reviewed articles is available in Tables, Figures, and Additional files.

## Appendix A

Search terms used in each database consulted

(1) web of science = (TI = (‘sedentary* behavior*’ OR ‘sedentary* behavior*’ OR sitting OR ‘watching TV’ OR ‘TV watching’ OR ‘TV viewing’ OR Television OR ‘video watching’ OR ‘watching video’ OR ‘computer use’ OR ‘internet use ‘OR ‘screen time’) AND TI = (China OR Chinese) = 329).

(2) PubMed = ((‘sedentary* behavior*’ [Title] OR ‘sedentary* behavior*’ [Title] OR sitting [Title] OR ‘watching TV’ [Title] OR ‘TV watching’ [Title] OR ‘TV viewing’ [Title] OR Television [Title] OR ‘video watching’ [Title] OR ‘watching video’ [Title] OR ‘computer use’ [Title] OR ‘internet use’ OR ‘screen time’ [Title])) AND (China OR Chinese [Title/Abstract]) = 82.

(3) EBSCO Academic Search Premier = (TI(‘sedentary behavior*’ OR ‘sedentary behavior*’ OR sitting OR ‘watching TV’ OR ‘TV watching’ OR ‘TV viewing’ OR Television OR ‘video watching’ OR ‘watching video’ OR ‘computer use’ OR ‘internet use’ OR ‘screen* time’) AND SU (China or Chinese) = 281).

(4) EBSCO SPORTDiscus = (TI(‘sedentary behavior*’ OR ‘sedentary behavior*’ OR ‘sitting time’ OR ‘watching TV’ OR ‘TV watching’ OR ‘TV viewing’ OR Television OR ‘video watching’ OR ‘watching video’ OR ‘computer use’ OR ‘internet use’ OR ‘screen* time’) AND SU (China or Chinese) = 20).

(5) EBSCO MEDLINE Complete = (TI(‘sedentary behavior*’ OR ‘sedentary behavior*’ OR ‘sitting time’ OR ‘watching TV’ OR ‘TV watching’ OR ‘TV viewing’ OR Television OR ‘video watching’ OR ‘watching video’ OR ‘computer use’ OR ‘internet use’ OR ‘screen* time’) AND SU (China or Chinese) = 118).

(6) Psychology and Behavioral Sciences Collection = (TI(‘sedentary behavior*’ OR ‘sedentary behavior*’ OR ‘sitting time’ OR ‘watching TV’ OR ‘TV watching’ OR ‘TV viewing’ OR Television OR ‘video watching’ OR ‘watching video’ OR ‘computer use’ OR ‘internet use’ OR ‘screen* time’) AND SU (China or Chinese) = 23).

(7) ScienceDirect = (TI(‘sedentary behavior*’ OR ‘sedentary behavior*’ OR ‘sitting time’ OR ‘watching TV’ OR ‘TV watching’ OR ‘TV viewing’ OR Television OR ‘video watching’ OR ‘watching video’ OR ‘computer use’ OR ‘internet use’ OR ‘screen* time’) AND SU (China or Chinese) = 41).

(8) ERIC = (TI(‘sedentary behavior*’ OR ‘sedentary behavior*’ OR ‘sitting time’ OR ‘watching TV’ OR ‘TV watching’ OR ‘TV viewing’ OR Television OR ‘video watching’ OR ‘watching video’ OR ‘computer use’ OR ‘internet use’ OR ‘screen* time’) AND SU (China or Chinese) = 36).

(9) PsycINFO = TI(‘sedentary behavior*’ OR ‘sedentary behavior*’ OR ‘sitting time’ OR ‘watching TV’ OR ‘TV watching’ OR ‘TV viewing’ OR Television OR ‘video watching’ OR ‘watching video’ OR ‘computer use’ OR ‘internet use’ OR ‘screen* time’) AND SU (China or Chinese) = 95).

(10) Scopus = (①TITLE (sedentary behavior* OR sedentary behavior*) AND (China OR Chinese) (LIMIT-TO (AFFILCOUNTRY, “China”)) AND (LIMIT-TO (LANGUAGE, “English”)) =55; ② TITLE (‘sitting AND time’) AND (China OR Chinese) AND (LIMIT-TO (AFFILCOUNTRY, “China”)) AND (LIMIT-TO (LANGUAGE, “English”)) =11; ③TITLE (watching AND tv’ OR ‘tv AND watching’) AND (China OR Chinese) AND (LIMIT-TO (AFFILCOUNTRY, “China”) OR LIMIT-TO (AFFILCOUNTRY, “Taiwan”)) AND (LIMIT-TO (LANGUAGE, “English”)) =11; TITLE (‘video AND watching’ OR ‘watching AND video’) AND (China OR Chinese) AND (LIMIT-TO (AFFILCOUNTRY, “China”) OR LIMIT-TO (AFFILCOUNTRY, “Hong Kong”)) AND (LIMIT-TO (LANGUAGE, “English”)) =26) = 103.

Chinese Records: ((11) CNKI + (12) WANGFANG DATA) = (Tittle = sedentary behavior = 31; Tittle = sitting with less move = 28; Tittle = sitting time = 3; Tittle = sitting lifestyle = 3; Tittle = sitting behavior = 11; Tittle = prolonged sitting behavior = 29; Tittle = prolonged sitting with less move = 5; Tittle = prolonged sitting with no move = 11; Tittle = prolonged sitting lifestyle = 4; Tittle = prolonged sitting time = 2; Tittle = screen time = 19; Tittle = television watching time = 29) = 175.
